# Genetic Deletion of *Hesx1* Promotes Exit from the Pluripotent State and Impairs Developmental Diapause

**DOI:** 10.1016/j.stemcr.2019.10.014

**Published:** 2019-11-21

**Authors:** Sara Pozzi, Sarah Bowling, John Apps, Joshua M. Brickman, Tristan A. Rodriguez, Juan Pedro Martinez-Barbera

**Affiliations:** 1 The Novo Nordisk Foundation Center for Stem Cell Biology – DanStem, University of Copenhagen, 3B Blegdamsvej, 2200 Copenhagen N, Denmark; 2 Developmental Biology and Cancer Programme, Birth Defects Research Centre, Great Ormond Street Institute of Child Health, University College London, London WC1N 1EH, UK; 3 Stem Cell Program, Boston Children's Hospital, Boston, MA 02115, USA; 4 Department of Stem Cell and Regenerative Biology, Harvard University, Cambridge, MA 02138, USA; 5 National Heart and Lung Institute, Imperial College London, London 6W3 6LY, UK

**Keywords:** Hesx1, pluripotency, diapause, embryonic stem cells, mouse development, LIF/STAT3 signaling, ERK pathway

## Abstract

The role of the homeobox transcriptional repressor HESX1 in embryonic stem cells (ESCs) remains mostly unknown. Here, we show that *Hesx1* is expressed in the preimplantation mouse embryo, where it is required during developmental diapause. Absence of *Hesx1* leads to reduced expression of epiblast and primitive endoderm determinants and failure of diapaused embryos to resume embryonic development after implantation. Genetic deletion of *Hesx1* impairs self-renewal and promotes differentiation toward epiblast by reducing the expression of pluripotency factors and decreasing the activity of LIF/STAT3 signaling. We reveal that *Hesx1-*deficient ESCs show elevated ERK pathway activation, resulting in accelerated differentiation toward primitive endoderm, which can be prevented by overexpression of *Hesx1*. Together, our data provide evidence for a novel role of *Hesx1* in the control of self-renewal and maintenance of the undifferentiated state in ESCs and mouse embryos.

## Highlights

• *Hesx1*-deficient embryos fail to reinitiate normal development after diapause
• *Hesx1* expression in ESCs is controlled by core pluripotency factors
• *Hesx1* regulates ESC differentiation toward epiblast and primitive endoderm
• *Hesx1* promotes LIF/STAT3 signaling and inhibits ERK signaling

## Teaser

Pozzi and colleagues have used *Hesx1*-defficient ESCs to reveal that *Hesx1* expression is regulated by intrinsic and extrinsic pluripotency-stimulating signals. They show that *Hesx1* promotes ESC self-renewal and inhibits ESC differentiation toward both the epiblast and primitive endoderm cell fates. They report the expression of *Hesx1* in preimplantation embryos, where it is required to resume normal embryonic development following diapause.

## Introduction

HESX1 (Homeobox gene expressed in stem cells 1) is a homeobox transcriptional repressor that was initially isolated from murine embryonic stem cells (ESCs) over 25 years ago (Thomas and Rathjen, 1992, Webb et al., 1993). *Hesx1* is crucial for proper forebrain and pituitary development in both mice (Andoniadou et al., 2007) and humans, and inactivating mutations in *HESX1* underlie septo-optic dysplasia (Dattani et al., 1998). It has been proposed that *HESX1* might be required for the maintenance of pluripotency in human ESCs (Richards et al., 2004), and more recently a beneficial role during somatic cell reprogramming has been demonstrated (Polo et al., 2012), suggesting a role for HESX1 in regulation of pluripotency. However, the consequences of the genetic deletion of *Hesx1* in ESCs have not been yet investigated. Here, we reveal a critical function of *Hesx1* in self-renewal and maintenance of the undifferentiated state in ESCs and mouse embryos.

## Results

### Loss of *Hesx1* Results in Failure to Resume Embryonic Development Following Diapause

**Figure 1:**
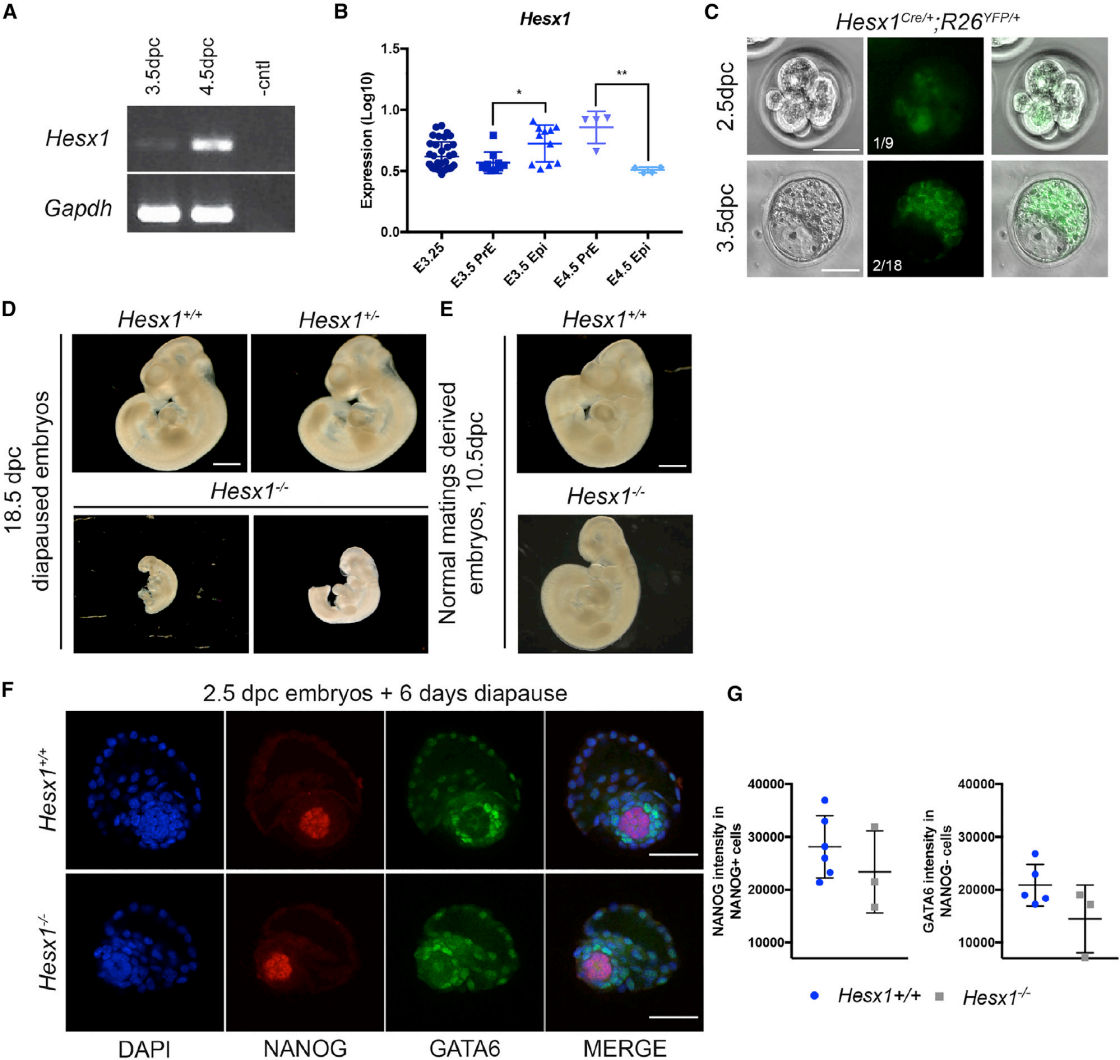
Lack of *Hesx1* Expression in Embryos Disrupts Developmental Diapause (A) *Hesx1* expression in 3.5- and 4.5-dpc C57BL/6J WT blastocysts. (B) *Hesx1* expression at different time points of preimplantation development measured by single-cell microarray. *Hesx1* is expressed at higher levels in the Epi lineage at 3.5 dpc (n = 10, ^∗^p < 0.01), but its expression becomes associated with PrE at 4.5 dpc (n = 4, ^∗∗^p < 0.005). (C) Bright-field and immunofluorescence images of 2.5- and 3.5-dpc *Hesx1*^*Cre/*+^;*R26*^*YFP/*+^ embryos. Only 1 of 9 and 2 of 18 embryos showed YFP expression. Scale bars, 50 μm. (D) 18.5-dpc diapaused embryos (8 days in diapause, then transferred to recipient females for further 10 days). Scale bar, 1 mm. (E) 10.5-dpc *Hesx1*^+*/*+^ and *Hesx1*^*−/−*^ embryos derived from conventional matings. Scale bar, 1 mm. (F) Immunofluorescence against NANOG and GATA6 in embryos after 6 days of diapause. Scale bar, 50 μm. (G) Scatterplot of NANOG and GATA6 mean fluorescence intensity in *Hesx1*^+*/*+^ (n = 6) and *Hesx1*^*−/−*^ (n = 3) embryos subjected to 6 days of diapause.

*Hesx1* expression in preimplantation embryos has not been reported (Thomas and Beddington, 1996). RT-PCR analysis revealed the presence of *Hesx1* mRNA in wild-type (WT) embryos (C57BL/6J background) at 3.5 and 4.5 days post coitum (dpc) (Figure 1A). Analysis of published data from single-cell microarray gene expression (Ohnishi et al., 2014) confirmed the expression of *Hesx1* from early (embryonic day 3.25 [E3.25]) to late blastocyst stages (E4.5) (Figure 1B). At later stages, *Hesx1* expression was detected in the primitive endoderm (PrE) (n = 4, p < 0.01). Immunofluorescence staining against GFP, to detect YFP expression, revealed the presence of YFP^+^ blastomeres in one of nine 8-cell stage morulas, two of 18 3.5-dpc blastocysts and none of nine 4.5-dpc blastocysts derived from *Hesx1*^*Cre/*+^;*R26*^*YFP/YFP*^ X *Hesx1*^+*/−*^ crosses (Figure 1C). YFP expression was restricted to cells in the inner cell mass (ICM), and no staining was observed in the trophoblast. The low proportion of embryos showing YFP expression is likely due to the loss of regulatory elements in the *Hesx1* locus caused by the targeting approach (i.e., introns 1–3 and exons 1–4 were replaced by a *Cre* cDNA [Andoniadou et al., 2007]).

A preimplantation phenotype for *Hesx1* mutants has not been previously described. However, the role of *Hesx1* in diapause has not been investigated, despite the conservation of the core transcriptional circuitry operating in the preimplantation epiblast (Epi) (Boroviak et al., 2015). To test the ability of diapaused embryos to resume development, we transferred a total of 81 blastocysts diapaused for 8 days directly into the uterus of pseudo-pregnant females and dissected the embryos 8 days later. At this time point, embryos were staged around 10.5 dpc, despite having being gestated for 18.5 days in total (Figure 1D). A total of 58 embryos were recovered, and genotyping analysis revealed 23 *Hesx1*^+*/*+^, 29 *Hesx1*^+*/−*^, and only 6 *Hesx1*^*−/−*^, therefore demonstrating a significant loss of *Hesx1*^*−/−*^ mutants and a strong deviation from the expected Mendelian ratios (Table S1; p = 0.0069). In addition to the expected forebrain defects (Andoniadou et al., 2007), diapaused mutant embryos displayed severe developmental delay and small size (Figure 1D). These defects have not been previously observed in *Hesx1*-deficient mutants derived from conventional matings (Figure 1E) (Andoniadou et al., 2007), suggesting an involvement of *Hesx1* in the maintenance of the expanded Epi when the preimplantation period is prolonged during diapause.

To further investigate failure in resuming development, we induced and maintained 2.5-dpc embryos from *Hesx1*^+*/−*^ intercrosses in a diapause state for 6 days. Diapaused blastocysts were then stained with antibodies against NANOG (Epi) and GATA6 (PrE), and the maximum fluorescence intensity (MFI) of the two markers was quantified. This analysis revealed a trend toward a reduction in the expression of both markers in *Hesx1*^*−/−*^ blastocysts (Figures 1F and 1G). Taken together, our results suggest that *Hesx1* is expressed at preimplantation stages, when it is required to maintain normal expression of NANOG and GATA6 and to resume embryonic development after implantation.

### *Hesx1* Expression Is Controlled by Intrinsic and Extrinsic Signals Associated with Maintenance of the Naive Pluripotent State

**Figure 2:**
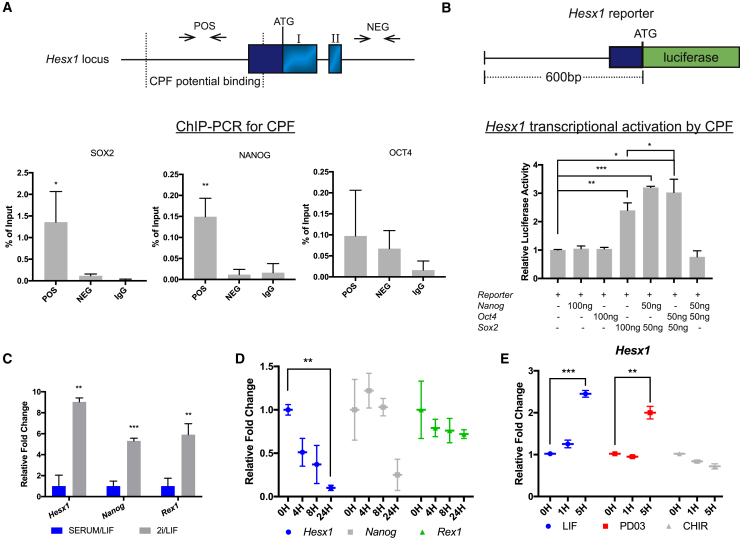
*Hesx1* Expression in ESCs Is Regulated by Naive Pluripotency-Promoting Signals (A) ChIP-PCR showing the binding of SOX2, NANOG, and OCT3/4 to the *Hesx1* promoter (n = 3 experiments). Diagram depicting part of the *Hesx1* locus, where POS is the region reported to be occupied by core pluripotency factors (CPF) and NEG represents a region in which binding was not reported. (B) Schematic diagram of the firefly luciferase reporter used in the co-transfection experiments in HEK-293T cells. Synergistic activation of *Hesx1* transcription by SOX2, NANOG, and OCT3/4 (n = 3 experiments). (C) qRT-PCR for *Hesx1*, *Nanog*, and *Rex1* expression in 2iLIF relative to serum/LIF (n = 3 experiments). (D) qRT-PCR for *Hesx1*, *Nanog*, and *Rex1* expression upon removal of the PD03 and CHIR (2i). Point 0H represents cells in 2i prior to inhibitor withdrawal (n = 3 experiments). (E) qRT-PCR showing that *Hesx1* expression is rapidly upregulated in WT ESCs upon LIF stimulation and MEK inhibition. Point 0H represents cells after a starvation period of 12 h in serum-free medium (n = 3 experiments).

The discovery of an early role for *Hesx1* in diapause prompted us to investigate whether *Hesx1* might regulate maintenance of the ESC state. Analysis of published chromatin immunoprecipitation sequencing (ChIP-seq) data (Marson et al., 2008) (Figure S1A) revealed the potential co-occupancy of different core pluripotency factors (CPFs) on the *Hesx1* promoter region. ChIP-qPCR on WT ESCs cultured in serum/leukemia inhibitory factor (LIF) revealed a significant enrichment in the amount of chromatin bound to SOX2 and NANOG and a non-significant increase in OCT3/4-bound chromatin (Figure 2A). To assess possible functional consequences, we carried out luciferase assays in HEK-293T cells. Co-transfection of a plasmid expressing SOX2 with a reporter containing a 600-bp region upstream of the ATG of the *Hesx1* locus, which includes the CPF binding sites, revealed a significant upregulation of luciferase activity relative to the controls (Figure 2B). Simultaneous co-transfection of plasmids expressing SOX2, NANOG, and OCT3/4 resulted in a further elevation of the luciferase activity compared with cells transfected only with SOX2 (Figure 2B). SOX2 binding sites are found in the *Hesx1* promoter (Eroshkin et al., 2002), but the elements bound to OCT4 and NANOG have not been described. The reasons underlying the lack of consensus sequences for OCT4 and NANOG are not known, despite it having been shown that both these factors are bound to the *Hesx1* promoter by ChIP-seq.

Naive pluripotency can be stimulated by culturing ESCs in defined conditions known as 2iLIF. This comprises inhibitors of mitogen-activated protein kinase (PD03) and glycogen synthase kinase 3 (CHIR), in addition to the cytokine LIF. These conditions stimulate the expression of factors involved in the maintenance of the naive state (Ying et al., 2008). Culture of WT ESCs in 2iLIF for 48 h followed by qRT-PCR analysis revealed a 9-fold increase *Hesx1* mRNA levels compared with serum/LIF conditions (Figure 2C). Conversely, *Hesx1* expression was downregulated 9-fold 24 h after removal of PD03 and CHIR (Figure 2D). The up- and downregulation in *Hesx1* expression was similar to that observed for *Nanog* and *Rex1* (Figures 2C and 2D). MEK inhibition alone, or 2i culture conditions, can increase expression of NANOG and other CPFs (i.e., OCT4) (Kim et al., 2014). To dissect the pathways regulating *Hesx1* expression in ESCs, we kept WT cells in N2B27 without LIF or inhibitors for 12 h, followed by addition of LIF, PD03, or CHIR. qRT-PCR at 1 and 5 h post supplementation revealed the upregulation of *Hesx1* expression upon addition of LIF and MEKi, which reached statistical significance after 5 h (Figure 2E). Together, these findings suggest that *Hesx1* expression is regulated by both external signaling pathways and CPFs, which together promote maintenance of ESC self-renewal and pluripotency.

### *Hesx1* Expression in ESCs Restricts Exit toward an Epi Fate by Reinforcing LIF Signaling

**Figure 3:**
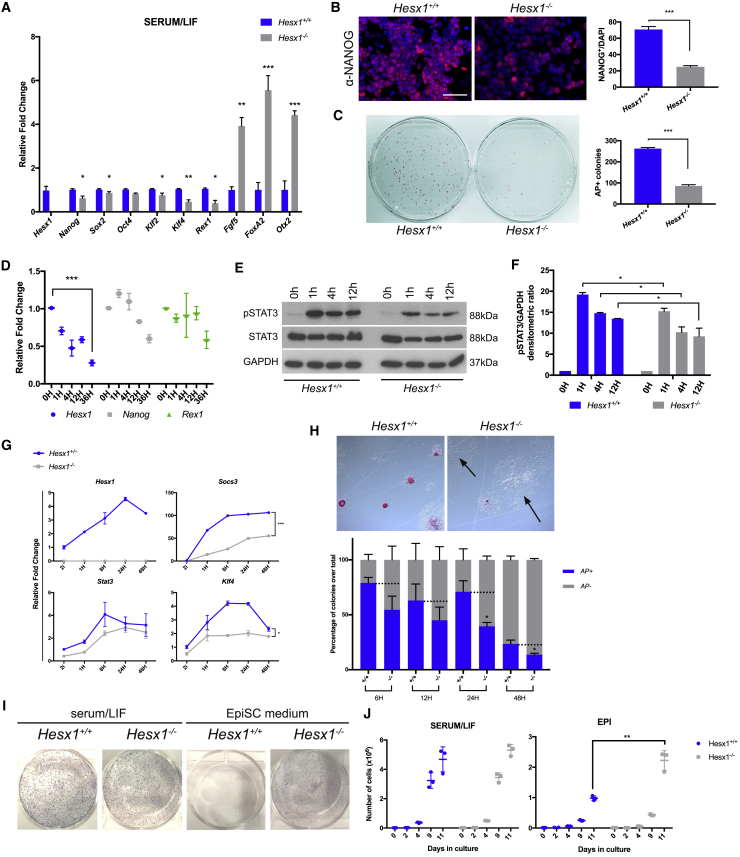
Loss of *Hesx1* in ESCs Leads to Reduced Response to LIF and Premature Epiblast Differentiation (A) qRT-PCR evaluating the levels of expression of both pluripotency and commitment-related factors (n = 3 experiments). (B) Immunofluorescence staining against NANOG showing a reduction in NANOG^+^ cells in *Hesx1*^*−/−*^ ESCs (n = 3 independent ESC clones). Scale bar, 50 μm. (C) AP staining after 6 days of culture at clonal density (n = 4 experiments using three ESC clones). (D) qRT-PCR showing that *Hesx1*, *Nanog*, and *Rex1* mRNA levels gradually decline when *Hesx1*^+*/*+^ ESCs are deprived of LIF (n = 3 ESC clones). (E and F) Western blot detection (E) and quantification (F) showing reduction of pSTAT3 in *Hesx1*^*−/−*^ mutant ESCs in response to LIF, with no difference in STAT3 (n = 3 ESC clones in three independent experiments). (G) qRT-PCR time course of ESCs cultured in 2i for three passages before addition of LIF (n = 3 ESC clones in three independent experiments). (H) AP staining for WT and *Hesx1*^*−/−*^ ESCs 24 h after LIF starvation. The majority of the *Hesx1*^*−/−*^ ESCs cannot retain pluripotency and generate large colonies of disperse flat cells (arrows). Quantification confirms a reduction in the number of AP^+^ colonies in *Hesx1*^*−/−*^ relative to *Hesx1*^+*/*+^ ESCs at all analyzed time points (n = 3 ESC clones and three independent experiments). (I) When cultured in EpiSC medium, *Hesx1*^*−/−*^ mutant ESCs generate a higher density of EpiSC colonies compared with *Hesx1*^+*/*+^ controls. In contrast, colony density is similar between genotypes when cells are cultured in serum/LIF. (J) Cellular growth rates in either serum/LIF or EpiSC medium (n = 3 ESC clones in three independent experiments).

To assess whether the lack of *Hesx1* in ESCs may have molecular consequences, we generated *Hesx1*-deficient clones by direct derivation of ESC lines from *Hesx1*-deficient (*Hesx1*^*−/−*^) and *Hesx1*^+*/*+^ control blastocysts. qRT-PCR detected the downregulation of pluripotency markers (i.e., *Nanog*, *Rex1*, *Klf4*) and upregulation of lineage commitment markers (i.e., *Fgf5*, *FoxA2*, *Otx2*) in *Hesx1*^*−/−*^ relative to *Hesx1*^+*/*+^ ESCs (Figure 3A). Immunostaining revealed a significant reduction in number of NANOG^+^ nuclei in *Hesx1*^*−/−*^ ESCs (Figure 3B). The ability to self-renew was evaluated by clonal assay followed by alkaline phosphatase (AP) staining. *Hesx1*^*−/−*^ ESCs showed a marked reduction in numbers of AP^+^ colonies after 6 days in culture compared with control cells (Figure 3C), suggesting that *Hesx1* is important for suppressing ESC differentiation.

*Hesx1* has been proposed to be a direct transcriptional target of LIF/STAT3 signaling (Martello et al., 2013), and analysis of STAT3 ChIP-seq data suggests a direct binding on the *Hesx1* promoter (Figure S1). In agreement with this notion, when *Hesx1*^+*/*+^ ESCs were cultured in serum/LIF and subsequently deprived of LIF signaling through removal of LIF in the medium, *Hesx1* as well as *Nanog* and *Rex1* expression were significantly reduced (*Hesx1*, 70%; *Nanog*, 40%; *Rex1*, 40%) (Figure 3D). We next sought to evaluate whether the ability to respond to LIF signaling may be impaired in *Hesx1*-deficient cells. To this end, *Hesx1*^+*/*+^ and *Hesx1*^*−/−*^ ESCs were cultured in serum-free medium for 12 h and subsequently subjected to LIF stimulation (Figures 3E and 3F). Western blot analyses revealed that while the amount of total STAT3 did not differ between genotypes (Figures 3E and 3F), levels of phospho-STAT3 were significantly reduced in *Hesx1*^*−/−*^ relative to *Hesx1*^+*/*+^ ESCs (Figures 3E and 3F). Next, we evaluated the response of *Hesx1*-deficient and WT ESCs to LIF stimulation in 2i conditions. STAT3 targets such as *Socs3*, *Klf4*, and *Stat3* were rapidly upregulated in *Hesx1*^+*/*+^ ESCs in response to LIF supplementation (Figure 3G). However, the transcriptional activation of these target genes was reduced in *Hesx1*^*−/−*^ relative to *Hesx1*^+*/*+^ ESCs (Figure 3G). Response to LIF was further evaluated in a LIF-withdrawal assay to assess the ability of ESCs to resist differentiation. In this assay, *Hesx1*^*−/−*^ ESCs showed reduced retention of AP activity when compared with *Hesx1*^+*/*+^ ESCs, as evidenced by the lower number of AP^+^ colonies. Conversely, numbers of AP^−^ colonies were increased in the absence of *Hesx1* (Figure 3H). Together, these data suggest that loss of *Hesx1* in ESCs results in reduced LIF signaling.

The transition from naive pluripotency to differentiated primed Epi state is associated with a loss in STAT3 responsiveness, which seems to be partly regulated by a reduction in pSTAT3 levels (Yang et al., 2010). We sought to explore whether *Hesx1*^*−/−*^ ESCs may be prompted to transit from naive to Epi state. *Hesx1*^+*/*+^ and *Hesx1*^*−/−*^ ESCs were cultured in either serum/LIF or in the presence of activin and fibroblast growth factor 2 (FGF2) to induce Epi stem cell (EpiSC) differentiation (Tesar et al., 2007). Growth analysis showed no apparent differences in total cell numbers between *Hesx1*-deficient and control cells when cultured in the presence of serum/LIF. In contrast, numbers of *Hesx1*^*−/−*^ cells were significantly increased in the presence of activin and FGF2 relative to the *Hesx1*^+*/*+^ control cells (Figure 3J). In accordance, numbers of EpiSC colonies were increased in the *Hesx1*^*−/−*^ relative to the *Hesx1*^+*/*+^ cell cultures, while both genotypes generated comparable ESC colony numbers in serum/LIF conditions (Figure 3I). Together, these analyses demonstrate that loss of *Hesx1* leads to decreased LIF/STAT3 signaling and elevated response to differentiation cues, suggesting that *Hesx1* is required to restrict exit from the naive state toward an Epi cell fate.

### *Hesx1* Expression in ESCs Limits Exit toward a PrE Fate by Inhibiting ERK Signaling

**Figure 4:**
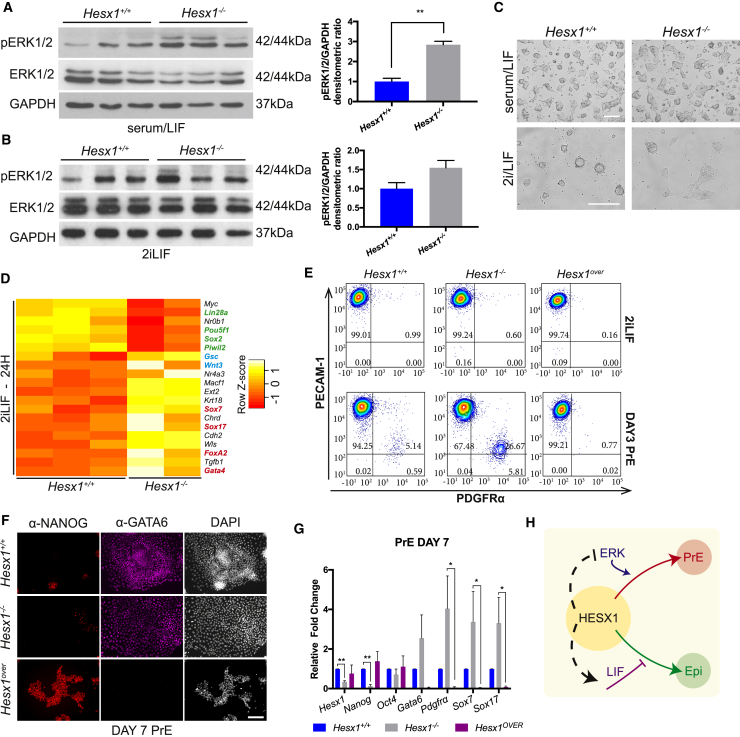
Loss of *Hesx1* in ESCs Results in Increased ERK Signaling and Promotion of the PrE Fate (A and B) Western blot for pERK1/2 and ERK1/2 on WT and *Hesx1*^*−/−*^ ESCs cultured in serum/LIF (A) and 2iLIF (B) conditions (n = 3 ESC clones in three independent experiments). (C) Bright-field images of *Hesx1*^+*/*+^ and *Hesx1*^*−/−*^ ESCs cultured in serum/LIF or 2iLIF. Scale bars, 200 μm. (D) Heatmap of the differentially expressed genes showing the lower expression of pluripotency-associated (green) and higher expression of differentiation (blue and red) markers in *Hesx1*^*−/−*^ relative to WT ESCs. Data represent the differentially expressed genes 24 h after 2i removal. (E) Flow-cytometry density plots of *Hesx1*^+*/*+^, *Hesx1*^*−/−*^, and *Hesx1*^*over*^ ESCs cultured in 2iLIF or after 3 days of PrE differentiation. Cells are stained with PECAM-1 (y axis) and PDGFRα (x axis). (F) Immunofluorescence staining for NANOG (red) and GATA6 (purple) on *Hesx1*^+*/*+^, *Hesx1*^*−/−*^, and *Hesx1*^*over*^ cells following 7 days of PrE differentiation. DNA is counterstained with DAPI. Scale bar, 100 μm. (G) qRT-PCR after 7 days of PrE differentiation (n = 3 ESC clones in three independent experiments). (H) Schematic depicting the summary of the findings. HESX1 promotes the undifferentiated state in ESCs by negatively regulating ERK activation and by reinforcing LIF/STAT3 signaling. Loss of *Hesx1* results in increased ERK and concomitant decreased in pSTAT3 signaling with a consequent premature differentiation toward endoderm and epiblast lineages, respectively.

FGF/ERK signaling is required to instruct the exit of ESCs from the self-renewal to initiate differentiation program (Kunath et al., 2007). Three *Hesx1*^+*/*+^ and three *Hesx1*^*−/−*^ ESC clones were cultured under self-renewing and ground-state conditions, and whole-cell lysates were collected to evaluate the levels of phosphorylation of ERK1/2 (Figure 4A). *Hesx1*^*−/−*^ ESCs showed increased levels of pERK1/2 compared with *Hesx1*^+*/*+^ controls (Figure 4A), both in serum/LIF and 2iLIF culture conditions (Figure 4B). Although ESCs from both genotypes formed tightly packed colonies in ground-state conditions, *Hesx1*^*−/−*^ ESCs showed a marked delay in acquiring this typical cellular morphology when switched from serum/LIF to 2iLIF medium (Figure 4C). As signaling downstream of pERK initiates epithelial-to-mesenchymal transition, these morphological defects are consistent with the elevated ERK activity observed in *Hesx1*^*−/−*^ ESCs.

To assess molecularly the ability of *Hesx1*^*−/−*^ ESCs to exit the naive state and differentiate, we performed RNA sequencing on *Hesx1*^+*/*+^ and *Hesx1*^*−/−*^ ESCs 24 h after 2iLIF removal from ground-state culture conditions (Figure 4D). Whole-transcriptome analysis revealed lower expression of pluripotency markers in *Hesx1*^*−/−*^ ESCs compared with WT controls (i.e., *Lin28a*, *Pou5f1*, *Sox2*, *Piwil2*). In addition, *Hesx1*^*−/−*^ ESCs showed higher expression of Epi (*Gsc*, *Wnt3a*) and PrE (*Sox7*, *Sox17*, *FoxA2*, *Gata4*) markers relative to control *Hesx1*^+*/*+^ ESCs (Figure 4D). Gene ontology term analysis of the top 500 up- and downregulated genes in *Hesx1*^*−/−*^ ESCs (log_2_ fold change >1 and <−1, respectively, with adjusted p value <0.05) revealed a significant enrichment of early developmental terms (i.e., Theiler stage 2–5), whereas genes upregulated in *Hesx1*^*−/−*^ ESCs belonged mostly to terms related to extraembryonic tissues and visceral organs (Figures S2A and S2B). GREAT (Genomic Regions Enrichment of Annotation Tool [McLean et al., 2010]) analysis of genes common between ERK targets (Hamilton and Brickman, 2014) and genes downregulated in *Hesx1*^*−/−*^ ESCs (Figure S3A) revealed a significant enrichment toward a negative regulation of both MAPK cascade activation and ERK protein phosphorylation (Figure S2C, see starred terms). For instance, negative regulators of this pathway such as *Dusp6*, *Dusp1*, *Spry1*, and *Spry4*, among others, were downregulated in *Hesx1*^*−/−*^ relative to *Hesx1*^+*/*+^ ESCs, suggesting that HESX1 might act as a negative regulator of MAPK signaling (Tables S2A and S2B).

The activation/phosphorylation of ERK has been shown to derepress PrE gene expression to promote functional priming toward this cell lineage (Hamilton and Brickman, 2014). As *Hesx1*^*−/−*^ ESCs showed increased pERK protein levels and upregulation of endodermal transcripts following exit from the naive state, we tested the ability of these mutant cells to undergo PrE differentiation *in vitro* (Anderson et al., 2017). We introduced *Hesx1*^over^ ESCs, which were generated by cloning a doxycycline-inducible *HA-*tagged*-HESX1* cDNA into ESCs, thus providing a *Hesx1* overexpression system (Figures S3A–S3C). During PrE differentiation (day 3), numbers of platelet-derived growth factor receptor α (PDGFRα; an endodermal marker) positive cells were approximately 27% in *Hesx1*^−/−^ ESCs (26.67% ± 5.61%) but around 5% in *Hesx1*^+*/*+^ ESCs (5.14% ± 0.59%) (Figure 4E). *Hesx1* overexpressing cells maintained an undifferentiated phenotype comparable with cells cultured in 2iLIF (Figure 4E), which resulted in a complete lack of PrE differentiation. In contrast, *Hesx1*^−/−^ ESC cells acquired a complete PrE phenotype, as demonstrated by GATA6 expression and loss of NANOG staining (Figure 4F). qRT-PCR analysis at day 7 of PrE differentiation revealed a downregulation of pluripotency-associated markers (*Nanog*, Oct4) and upregulation of endodermal transcripts (*Gata6*, *Pdgfrα*) in *Hesx1*^−/−^ ESCs relative to *Hesx1*^+*/*+^ control cells. In contrast, *Hesx1*^*over*^ ESCs showed a complete lack of PrE markers expression at day 7 (Figure 4G). These results suggest that *Hesx1* expression in naive ESCs prevents their differentiation into PrE by negatively regulating the activation of ERK signaling.

Collectively, this study demonstrates that *Hesx1* is a novel factor involved in the regulation of ESC fate (Figure 4H). We show that *Hesx1* expression responds to both intrinsic and extrinsic pluripotency-stimulating signals to promote self-renewal and inhibit differentiation toward both the Epi and PrE cell fates. Consistent with the *in vitro* data, we reveal that *Hesx1* is expressed in the ICM of the preimplantation mouse embryo, where it is required to resume development following diapause.

## Experimental Procedures

### Cell Culture

ESCs were cultured as described in Supplemental Experimental Procedures. Differentiation of ESC to EpiSC or PrE followed standard procedures (Tesar et al., 2007, Anderson et al., 2017; Supplemental Experimental Procedures).

### ESC Derivation

*Hesx1*^+*/*+^, *Hesx1*^+*/−*^, *Hesx1*^*−/−*^, *Hesx1*^*Cre/*+^;*R26*^*YFP/*+^, *Hesx1*^*Cre/*−^;*R26*^*YFP/*+^, and *Hesx1*^+*/*+^;*R26*^*YFP/*+^ ESCs were derived from breeding of mice previously described. *Hesx1*^+*/*+^ and *Hesx1* ^*−/−*^ ESCs were derived from *Hesx1*^+*/−*^ intercrosses (129/Sv background [Dattani et al., 1998]), *Hesx1*^*Cre/*+^;*R26*^*YFP/*+^, *Hesx1*^*Cre/*−^;*R26*^*YFP/*+^, and *Hesx1*^+*/*+^;*R26*^*YFP/*+^ ESCs from differential crosses between *Hesx1*^+*/−*^ (see above), *Hesx1*^*Cre/*+^ (C57BL/6J background [Andoniadou et al., 2007]), and *Hesx1*^+*/*+^; *R26*^*YFP/YFP*^ mice (C57BL/6J background). For ESC isolation, 3.5-dpc embryos were cultured on a layer of mitotically inactive mouse embryonic fibroblasts in N2B27 supplemented with 2iLIF.

### ChIP-PCR

Chromatin immunoprecipitation was adapted from the Young lab (Marson et al., 2008). Primers used to screen chromatin enrichment are listed in Table S3. Enrichment values were calculated using the percent input method (100 × 2ˆ(adjusted input − Ct(IP))).

### Dual-Luciferase Assay

Cells were lysed and analyzed for luciferase activity at 48 h post transfection. Total amount of transfected DNA was normalized by addition of an empty vector (pBluescript). Cells were harvested and assayed for luciferase activity using the Dual Luciferase Reporter Assay System (Promega) and luminescence was measured using a BMG FLUOstar Optima multiplate reader (BMG Labtech). More details can be found in Supplemental Experimental Procedures.

### Immunofluorescence Staining, Western Blot, and Flow Cytometry

See Supplemental Experimental Procedures for details. Quantification of NANOG and GATA6 levels was carried out by manually drawing a line through the nucleus of each cell (either NANOG^+^/GATA6^−^ or NANOG^−^/GATA6^+^) and measuring the MFI of NANOG or GATA6 across the line. For flow cytometry, cells were dissociated to a single-cell suspension and resuspended in FACS buffer (PBS + 10% fetal calf serum). Cells were then stained with DAPI to eliminate dead cells and gated using SSC-H against SSC-W to exclude doublets. Samples were processed on a BD Fortessa (BD Bioscience) and data analyzed using FCS Express. A list of antibodies used can be found in Table S4.

### qRT-PCR

Total RNA from cells or embryos was extracted using RNeasy micro or mini kits (Qiagen). Reverse transcription was performed using the iScript cDNA synthesis kit. Real-time PCR was performed using an iTaq Universal SYBR Green Supermix (Bio-Rad). Gene expression was calculated relative to *Gapdh* via the ΔΔCt method. A list of primers used can be found in Table S3.

### Diapause Induction

For induction of diapause (MacLean Hunter and Evans, 1998), 2.5-dpc *Hesx1*^+*/−*^ pregnant females from *Hesx1*^+*/−*^ intercrosses were injected intraperitoneally with 10 μg of tamoxifen (Sigma) and 3 mg of Depo-Provera (Pfizer). Diapaused blastocysts were surgically transferred into the uteri of 2.5-dpc pseudo-pregnant CD1 recipient females and embryos collected at the specific developmental stages. All mouse experiments were performed under the umbrella of a UK Home Office Project Licence and by trained Personal Licence holders.

### Statistical Analysis

Unless stated, all experiments were carried out on biological and experimental triplicates. All data represent mean ± SEM. Statistical significance was calculated with Student's t test.

## Author Contributions

S.P. and J.P.M.-B. conceived and designed the study, S.P. carried out the majority of the experiments. S.B. performed experiments. J.A. performed the bioinformatics analysis. T.A.R. and J.M.B. helped with experimental design and data interpretation. S.P. and J.P.M.-B. wrote the manuscript.

## Accession Numbers

The accession number for the RNA-sequencing data is ArrayExpress: E-MTAB-5816.

## Supplemental Information

Document S1. Supplemental Experimental Procedures, Figures S1–S3, and Tables S1, S3, and S4

Table S2. Gene Expression Data Comparing *Hesx1^−^*^*/^−^*^ Mutant versus *Hesx1*^*+/+*^ Wild-Type ES Cells

Document S2. Article plus Supplemental Information
