# Supplementary material for: Genetic Deletion of Hesx1 Promotes Exit from the Pluripotent State and Impairs Developmental Diapause
Source: Stem Cell Reports. 2019 Nov 21;13(6):970–9. doi: 10.1016/j.stemcr.2019.10.014 (PMC6915801; doi:10.1016/j.stemcr.2019.10.014)
Supplement: Document S1. Supplemental Experimental Procedures, Figures S1–S3, and Tables S1, S3, and S4 [file mmc1.pdf]

Stem Cell Reports, Volume *13*

Supplemental Information

Genetic Deletion of *Hesx1* Promotes Exit from the Pluripotent State and Impairs Developmental Diapause

Sara Pozzi, Sarah Bowling, John Apps, Joshua M. Brickman, Tristan A. Rodriguez, and Juan Pedro Martinez-Barbera

A

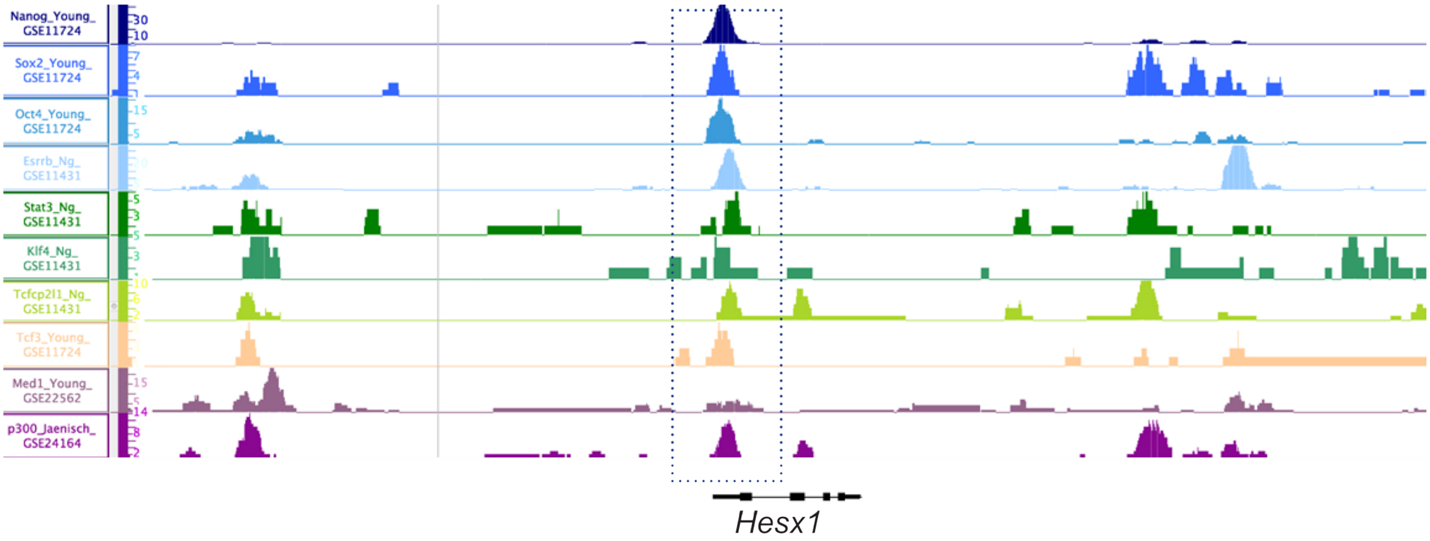

Nanog_Young_
GSE11724
Sox2_Young_
GSE11724
Oct4_Young_
GSE11724
Esrrb_Ng_
GSE11431
Stat3_Ng_
GSE11431
Klf4_Ng_
GSE11431
Tcfcp2l1_Ng_
GSE11431
Tcf3_Young_
GSE11724
Med1_Young_
GSE22562
p300_Jaenisch_
GSE24164
Hesx1

Figure S1.

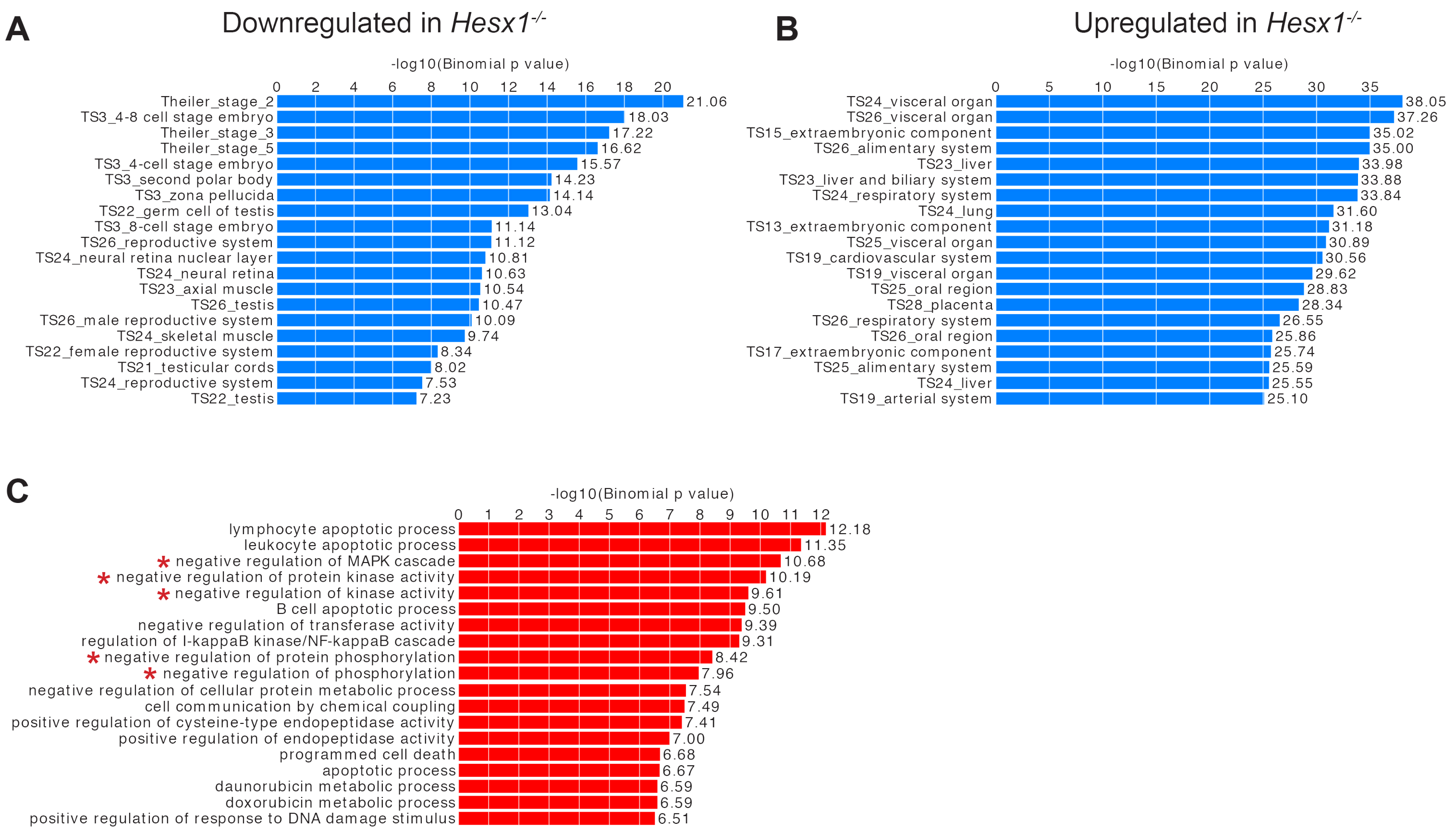
A
Downregulated in Hesx1-/-
-log10(Binomial p value)
0 2 4 6 8 10 12 14 16 18 20
Theiler_stage_2 21.06
TS3_4-8 cell stage embryo 18.03
Theiler_stage_3 17.22
Theiler_stage_5 16.62
TS3_4-cell stage embryo 15.57
TS3_second polar body 14.23
TS3_zona pellucida 14.14
TS22_germ cell of testis 13.04
TS3_8-cell stage embryo 11.14
TS26_reproductive system 11.12
TS24_neural retina nuclear layer 10.81
TS24_neural retina 10.63
TS23_axial muscle 10.54
TS26_testis 10.47
TS26_male reproductive system 10.09
TS24_skeletal muscle 9.74
TS22_female reproductive system 8.34
TS21_testicular cords 8.02
TS24_reproductive system 7.53
TS22_testis 7.23
B
Upregulated in Hesx1-/-
-log10(Binomial p value)
0 5 10 15 20 25 30 35
TS24_visceral organ 38.05
TS26_visceral organ 37.26
TS15_extraembryonic component 35.02
TS26_alimentary system 35.00
TS23_liver 33.98
TS23_liver and biliary system 33.88
TS24_respiratory system 33.84
TS24_lung 31.60
TS13_extraembryonic component 31.18
TS25_visceral organ 30.89
TS19_cardiovascular system 30.56
TS19_visceral organ 29.62
TS25_oral region 28.83
TS28_placenta 28.34
TS26_respiratory system 26.55
TS26_oral region 25.86
TS17_extraembryonic component 25.74
TS25_alimentary system 25.59
TS24_liver 25.55
TS19_arterial system 25.10
C
-log10(Binomial p value)
0 1 2 3 4 5 6 7 8 9 10 11 12
lymphocyte apoptotic process 12.18
leukocyte apoptotic process 11.35
* negative regulation of MAPK cascade 10.68
* negative regulation of protein kinase activity 10.19
* negative regulation of kinase activity 9.61
B cell apoptotic process 9.50
negative regulation of transferase activity 9.39
regulation of I-kappaB kinase/NF-kappaB cascade 9.31
* negative regulation of protein phosphorylation 8.42
* negative regulation of phosphorylation 7.96
negative regulation of cellular protein metabolic process 7.54
cell communication by chemical coupling 7.49
positive regulation of cysteine-type endopeptidase activity 7.41
positive regulation of endopeptidase activity 7.00
programmed cell death 6.68
apoptotic process 6.67
daunorubicin metabolic process 6.59
doxorubicin metabolic process 6.59
positive regulation of response to DNA damage stimulus 6.51

Figure S2.

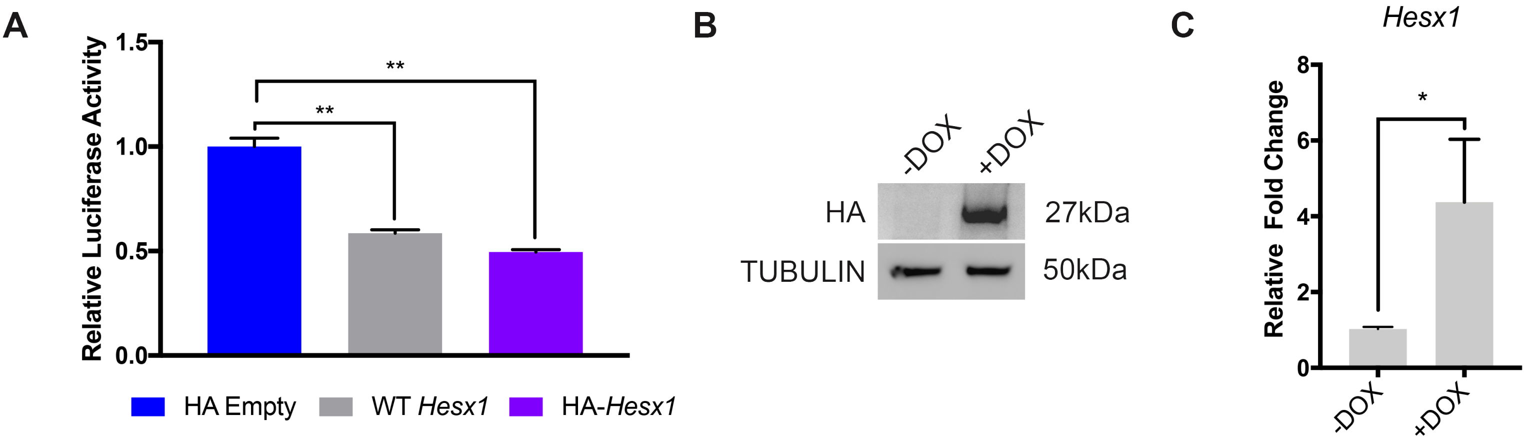
A
Relative Luciferase Activity
1.5
1.0
0.5
0.0
**
**
HA Empty
WT Hesx1
HA-Hesx1
B
-DOX
+DOX
HA
27kDa
TUBULIN
50kDa
C
Hesx1
Relative Fold Change
8
6
4
2
0
*
-DOX
+DOX

Figure S3.

Supplemental Figure legends

Figure S1. Multiple naïve state-associated factors display binding signal on the *Hesx1* promoter region. Related to Figure 2.

(A) UCSG Genome Browser snapshot (mm9, chr14) corresponding to a 16 kb region centred around the *Hesx1* locus. Dotted blue box encompass peaks falling into the region evaluated in Figure 2 B. From top to bottom, tracks represented are: GSE11724_NANOG, SOX2, OCT4, TCF3 (TCF7L1); GSE11431_ESSRB, STAT3, KLF4, TCFCP21L; GSE22563_MED1 and GSE24164_p300.

Figure S2. Doxycycline-inducible expression of HA-HESX1. Related to Figure 4.

(A) Dual luciferase assay showing relative levels of luciferase activity in CHO cells co-transfected with equal amounts of a *SV40-luciferase* reporter plus 100ng of *HA-empty*, *wild-type (WT)-Hesx1* and *HA-Hesx1* constructs. Transfection with the HA-tagged version of *Hesx1* leads to comparable levels of repression of luciferase activity compared to the WT construct, demonstrating that the HA tag does not interfere with the repressing activity of HESX1, n=3 experiments. **(B)** Western blot analysis against HA on *Hesx1^{over}* ES cells before and after induction with 100ng of Doxycycline. TUBULIN was used as a loading control. **(C)** qRT-PCR for *Hesx1* levels of expression in ES cells before and after DOX induction, showing *Hesx1* overexpression; n=3 experiments.

Figure S3. Analysis of transcriptomic data comparing *Hesx1$^{+/+}$* and *Hesx1$^{-/-}$* ES cells 24 hours after 2i removal. Related to Figure 4.

(A) MGI expression data on the top 500 downregulated genes (*padj*<0.05, $\log_2$FC<-1) in *Hesx1$^{-/-}$* ES cells showing enrichment for early developmental time points (i.e. 4-8 cell stage, Theiler stage 2, 3, 5). **(B)** MGI expression data on the top 500 upregulated genes (*padj*<0.05, $\log_2$FC>1) in *Hesx1$^{-/-}$* ES cells showing enrichment for endodermal tissues (i.e. extraembryonic components, liver). **(C)** GREAT analysis of biological processes enriched in a dataset composed by genes in common (n=191) between ERK transcriptional targets (Hamilton and Brickman, 2014) and genes downregulated in *Hesx1$^{-/-}$* ES cells (Figure S3 A). Red stars highlight processes linked to MAPK signalling, protein phosphorylation and kinase activity.

Table S1. Analysis of Mendelian ratios of diapaused embryos. Related to Figure 1.

	Expected proportions	Observed proportions
Hesx1$^{+/+}$	25%	40% (n=23)
Hesx1$^{+/-}$	50%	50% (n=29)
Hesx1$^{-/-}$	25%	10% (n=6)
**Total number of embryos**		58

**Chi-square test**	
**Chi-square**	9.966
**Degree of freedom (DF)**	2
***p* value (two-tailed)**	0.0069 (**)

Table S3. List of primers used for qRT-PCR and ChIP-PCR analysis. Related to Figures 1-4.

Marker	Forward primer (5'-3')	Reverse primer (5'-3')
Hesx1	GATCTTCCCAGTGAGACTTC	CAGGGTAGCAGTTCACTCTA
Nanog	CCTCCAGCAGATGCAAGAA	GCTTGCACTTCATCCTTTGG
Rex1	CGCTGTGGGCATTAGGTAAG	GCACACTCACTCTATTGAGAGAAGAA
Sox2	AAGGGTTCTTGCTGGGTTTT	AGACCACGAAAACGGTCTTG
Oct4	AAGCCCTCCCTACAGCAGAT	CTGGGAAAGGTGTCCCTGTA
Klf2	GCCTGTGGGTTCGCTATAAA	GATGTGGCTGACCATTCCTT
Klf4	CCAGCAAGTCAGCTTGTGAA	AATCCAACTTGAACATGCCC
Fgf5	AAAACCTGGTGCACCCTAGA	CATCACATTCCCGAATTAAGC
FoxA2	GGTCTCTACAGCAAGATGAATGG	TGGCACAGGACAGTCCAAG
Otx2	GACCCGGTACCCAGACATC	GCTCTTCGATTCTTAAACCATAC
T	GCTTCAAGGAGCTAACTAACGAG	GCCATGTACTCTTTCTTGCTGG
Socs3	GAGATTTCGCTTCGGGACTA	CTCCAAAAGCGAGTACCAGC
Stat3	GGAAATAACGGTGAAGGTGCT	CATGTCAAACGTGAGCGACT
Gata6	GGTCTCTACAGCAAGATGAATGG	TGGCACAGGACAGTCCAAG
Pdgfrα	AAGACCTGGGCAAGAGGAAC	GAACCTGTCTCGATGGCACT
Sox7	CTGCCTCATCCACATAGGG	CGGAGCTCAGCAAGATGC
Sox17	AGCCATTTCCTCCGTGGTGT	AACACTGCTTCTGGCCCTCAG
Gapdh	ATGACATCAAGAAGGTGGTG	CATACCAGGAAATGAGCTTG
POS	TTCGTGCACCACTTCTTCTG	CCAGCGGCTTAAGGAGTTAAT
NEG	TAAGCTCAGCTGTGGTGGCTG	GGCTGTCCTGGAACTCACTC

Table S4. List of antibodies used for IF and WB analysis. Related to Figures 1, 3 and 4.

Antibody	Vendor	Code	WB	IF	Cytometry
NANOG	eBioscience	14-5761	1:500	1:200	
OCT4	SantaCruz	Sc-5279	1:200	1:500	
SOX2	Abcam	Ab97959	1:200	1:200	
pSTAT3	Cell Signalling	9145	1:2000		
STAT3	Cell Signalling	4904	1:2000		
P-p42/44 MAPK	Cell Signalling	4370	1:1000		
P42/44 MAPK	Cell Signalling	4695	1:1000		
HA	Roche	12013819001	1:1000		
GAPDH	Millipore	MAB974	1:20000		
TUBULIN	Abcam	Ab6160	1:20000		
GFP	Abcam	Ab13970		1:2000	
GATA6	Cell Signalling	5851		1:200	
PECAM-1	BD Pharmingen	551262			1:200
PDGFRα	eBioscience	12-1401			1:200

Analysis of *Hesx1* expression at pre-implantation stages (Relative to Figure 1B)
Row values for *Hesx1* expression have been collected from single-cell microarray data (Ohnishi et al., 2014) on a total of 66 cells (36 cells from 6 embryos at E3.25, 22 cells from 3 embryos at E3.5 and 8 cells from 1 embryo at E4.5). The graph and the statistical analysis (Student's *t*-test) were produced using the Prism7 software.

Cell culture
ESCs were cultured on 0.1% gelatine coated plates in DMEM with 15% FCS (PAA), 2mM glutaMAX, 1% NEAA, 0.1mM 2-mercaptoethanol (Invitrogen) and 1000U/mL LIF (Millipore). For naïve conditions, cells were grown in N2B27 (made in house) with 1µM PD035901 and 3µM CHIR99021 (Medchem). For ES to EpiSC transition, $0.8x10^5$ cells were plated on fibronectin-coated wells in serum/LIF overnight. Medium was then changed to EpiSC medium with 12ng/mL FGF2 (R&D) and 10ng/mL Activin A (Peprotech). For PrE differentiation, endoderm base media with B27 minus insulin (Gibco), 20ng/mL Activin A, 3µM CHIR99021 and LIF was used. HEK and CHO cells were maintained in DMEM with 10% FCS.

Immunofluorescence staining, western blot and flow cytometry.
For IF, cells were fixed in 4% PFA and permeabilised in 0.1% PBST. Following O/N incubation at 4ºC with a primary antibody, cells were subjected to secondary antibody incubation for 1 hour at room temperature. Nuclei were visualised with DAPI (10µg/mL, Sigma). For WB, whole cell lysates were extracted with Basic Cell Lysis Solution (50mM Tris-Base pH 7.6, 150mM NaCl, 1% Triton X100) supplemented with a Protease Inhibitor Cocktail (Roche). WB was carried out as previously described (Mahmood and Yang, 2012). Alkaline phosphatase (AP) was detected using the Leukocyte Alkaline Phosphatase kit (Sigma). IF of embryos was carried out as previously described (Nichols and Smith, 2009). Embryos were fixed in 4% PFA in 0.01% PBST. After permeabilization, embryos were blocked in 2% horse serum in 0.1% PBST. Primary antibodies were incubated overnight at 4ºC. Secondary antibodies were applied for 1 hour at 4ºC. All imaging was performed on a Zeiss LSM-780 confocal microscope.

References
Ohnishi, Y., Huber, W., Tsumura, A., Kang, M., Xenopoulos, P., Kurimoto, K., Oleś, A.K., Araúzo-Bravo, M.J., Saitou, M., Hadjantonakis, A.-K., et al. (2014). Cell-to-cell expression variability followed by signal reinforcement progressively segregates early mouse lineages. Nat. Cell Biol. *16*, 27–37.
Mahmood, T., and Yang, P.-C. (2012). Western Blot: Technique, Theory, and Trouble Shooting. N. Am. J. Med. Sci. *4*, 429–434.
Nichols, J., and Smith, A. (2009). Naive and Primed Pluripotent States. Cell Stem Cell *4*, 487–492.
